# Associations of Forest Type, Parasitism and Body Condition of Two European Passerines, *Fringilla coelebs* and *Sylvia atricapilla*

**DOI:** 10.1371/journal.pone.0081395

**Published:** 2013-12-05

**Authors:** Bruntje Lüdtke, Isabelle Moser, Diego Santiago-Alarcon, Markus Fischer, Elisabeth KV. Kalko, H. Martin Schaefer, Marcela Suarez-Rubio, Marco Tschapka, Swen C. Renner

**Affiliations:** 1 Institute of Experimental Ecology, University of Ulm, Ulm, Germany; 2 Institute for Zoology, University of Freiburg, Freiburg, Germany; 3 Eberhard-Karls-Universität Tübingen, Tübingen, Germany; 4 Instituto de Ecología, Asociación Civil Xalapa, Veracruz, México; 5 Institute of Plant Sciences and Botanical Garden, University of Bern, Bern, Switzerland; 6 Institute of Experimental Ecology, University of Ulm, Ulm, Germany; 7 Smithsonian Tropical Research Institute, Bilbao, Panama; 8 Institute of Zoology, University of Natural Resources and Life Sciences, Vienna, Austria; 9 Smithsonian Conservation Biology Center, National Zoological Park, Front Royal, Virginia, United States of America; Hungarian Academy of Sciences, Hungary

## Abstract

Human-induced forest modification can alter parasite-host interactions and might change the persistence of host populations. We captured individuals of two widespread European passerines (*Fringilla coelebs* and *Sylvia atricapilla*) in southwestern Germany to disentangle the associations of forest types and parasitism by haemosporidian parasites on the body condition of birds. We compared parasite prevalence and parasite intensity, fluctuating asymmetries, leukocyte numbers, and the heterophil to lymphocyte ratio (H/L-ratio) among individuals from beech, mixed-deciduous and spruce forest stands. Based on the biology of bird species, we expected to find fewer infected individuals in beech or mixed-deciduous than in spruce forest stands. We found the highest parasite prevalence and intensity in beech forests for *F*. *coelebs*. Although, we found the highest prevalence in spruce forests for *S. atricapilla*, the highest intensity was detected in beech forests, partially supporting our hypothesis. Other body condition or health status metrics, such as the heterophil to lymphocyte ratio (H/L-ratio), revealed only slight differences between bird populations inhabiting the three different forest types, with the highest values in spruce for *F. coelebs* and in mixed-deciduous forests for *S. atricapilla*. A comparison of parasitized versus non-parasitized individuals suggests that parasite infection increased the immune response of a bird, which was detectable as high H/L-ratio. Higher infections with blood parasites for *S*. *atricapilla* in spruce forest indicate that this forest type might be a less suitable habitat than beech and mixed-deciduous forests, whereas beech forests seem to be a suboptimal habitat regarding parasitism for *F. coelebs*.

## Introduction

Animals interact with their environment in complex ways and can respond, for example, to changes in forest structure and parasite infections [1]–[3]. However, whether animals select a habitat influenced by intra- and inter-specific interactions [4], [5], such as parasitism, is not yet fully understood. In birds, parasites can increase mortality [6]–[8], decrease fecundity, or inhibit growth [9]. Indirect effects of parasites include altering habitat selection processes of hosts [10], modifying coexistence of species (e.g., [3]), changing host behavior [11], or structuring of animal communities [12].

In particular, newly introduced parasites are of relevance for parasite-naïve hosts due to a lack of co-adaptation between them [13]. In the case of avian haemosporidians, one serious pathological consequence is the destruction and active removal of infected erythrocytes, which may cause anemia in some individuals [14]. High prevalence of parasites in a given habitat can therefore result in acute infections and strong immunoreactions of infected individuals. Costs associated with parasitism could drive birds away from places with high infection risk; however, the role of forest types on host-parasite interactions is not yet rigorously investigated.

The history of forest structure modification by humans is extensive [15]. In the 19^th^ Century, when fossil fuels replaced wood as an energy resource, demand for timber as construction material increased [16], and this resulted in forest stands being dominated by *Picea abies* (spruce) in Germany [17]. Starting in the second half of the 20^th^ Century, forestry strategies changed toward more sustainable practices. Tree species composition and age class forests shifted back to more natural mixed-deciduous forests [17]. These changes in forest management also modified living conditions for birds, including changes in resource availability, e.g., food, foraging, and nesting sites [18], [19], and alterations of interspecific interactions [20].

Interspecific interactions such as parasitism should affect habitat selection and habitat quality. Habitat selection indicates habitat preference but not habitat quality per se [21]. Under natural conditions, habitat selection by birds should enhance fitness in preferred habitats [22], [23]. However, habitat selection in human-altered environments might be misleading, because cues for ‘good-quality habitat’, such as access to food, nesting sites, and refuge from predators, are obscured by human activities, e.g., by logging [23], [24]. Therefore, the assessment of factors that affect habitat selection should include an analysis of consequences of parasites on the hosts' body condition [23] and indirectly on hosts' habitat selection. Here, we investigate effects of infection by haemosporidian parasites on birds' body condition in different habitats and use several body condition measures as an indirect measure of habitat quality.

Parasitism can affect body condition parameters of birds [8] and is also linked to environmental conditions [25]. Because body condition is a traceable parameter [26], we can identify correlations between parasitism, forest type, and health components [21], [27]–[29]. Increased parasitism and decreased body condition attributable to changes in land-use intensity and forest management regimes can cause stress in birds [27]–[29]. Stress levels can be determined by measuring stress hormones and/or by measuring a section of the immune system, e.g., white blood cell counts, because leukocytes are an integral part of the immune defense and are related to stress [30]. The heterophil to lymphocyte ratio (H/L-ratio) is especially suitable for measuring chronic stress [30]–[36]. Therefore, the H/L-ratio can be used as surrogate of chronic stress in birds and should indirectly indicate the quality of various forest types for birds in reference to parasitism.

Haemosporidian parasites are transmitted by dipteran vectors. Avian haemosporidians have complex life cycles and need a dipteran definitive host –where they reproduce sexually– and an avian intermediate host to complete their life cycle [37]. Dipteran definitive hosts are more drastically affected by environmental conditions compared with vertebrate intermediate hosts [37]. Thus, changes in abiotic conditions can alter infection dynamics, which subsequently can increase or decrease the prevalence and the geographic range of both parasites and vectors [38]–[40]. Some Diptera vector families reproduce in standing water bodies (e.g., Culicidae) or require high humidity levels (e.g., Ceratopogonidae) to continue their life cycle [41]. Puddles on soft skid trails and forest ground are frequently formed by the use of heavy machinery during logging. Puddles on the ground can serve as nursery habitats for insect vectors, increasing their abundance and consequently infection risk [40], [42]. Hence, infection risk can vary according to forest type and forestry practices.

Here, we investigate the associations between forest types, haemosporidian parasitism, and body condition for two common and highly abundant European passerines (*Fringilla coelebs* European Chaffinch, and *Sylvia atricapilla* Blackcap). To our knowledge, this is the first attempt to link forest management to health parameters of bird species in Germany. Specifically, we hypothesize:

(1) Parasite prevalence and parasite intensity are higher in *F. coelebs* and *S. atricapilla* inhabiting spruce compared with beech or mixed-deciduous forest types.

Both bird species prefer mixed-deciduous forest types with a diversified understory and shrub layer [43], with a high structural and functional diversity of plants and insects [44]–[47]. Spruce forest should offer less optimal conditions for *F. coelebs* and *S. atricapilla* [43]. Birds inhabiting suboptimal spruce forest (territory size of the two bird species are larger in spruce forests, indicating that resources are distributed over larger areas, [43]) need to invest more energy and time in foraging, which can compromise a proper immune defense [48]. This might lead to higher parasite prevalence and parasite intensity of birds inhabiting spruce forests.

(2) Body condition indices and other health status metrics (fluctuating asymmetry, leukocyte numbers, and H/L-ratio) are indicative of suboptimal habitat conditions. We predict high asymmetry and high leukocyte counts and H/L-ratio in spruce forest and lower values in beech or mixed-deciduous forest stands (i.e., higher asymmetry, leukocyte numbers, and H/L-ratio for parasitized birds in spruce forest). In addition, we predict a higher H/L-ratio in infected than in uninfected birds, regardless of forest type. The H/L-ratio increases in infected *F. coelebs* and *S. atricapilla* because individuals build up a defense against parasites [8], [30]. Stressors in a habitat with food shortage [44]–[47] can lead to a decreased condition in birds. This situation can make them vulnerable to infection, and if previously infected, birds would have to use available resources and body reserves to mount an immune response [21]. When energy resources are used to fight off an infection, we expect organisms to have reduced investment in other life history traits [48], [49]. Hence, fluctuating asymmetry should increase during development of the feathers or extremities (e.g. [50]) because individuals face developmental challenges [51].

## Methods and Study Site

Capturing and handling birds as well as collecting blood were performed in compliance with federal and state laws. All permits were granted by the “*Regierungspräsidium Tübingen, Referat Artenschutz, Tierschutz*” (*RPT Tierversuch-Nr. 1056*). All birds were handled to best practice following the guidelines of the bird banding laboratory “*Vogelwarte Radolfzell*”. These guidelines on bird handling for scientific purpose implemented all steps requested by the animal welfare of the European Commission, which are implemented in the federal and state laws of Germany.

Assessment of Institutional Animal Care and Use Committee (IACUC) is part of the permit procedure; the state environmental offices of Baden-Württemberg (“*Regierungspräsidium Tübingen, Referat Artenschutz, Tierschutz*”) approved the study in 2011. The mandatory training of the field workers was assessed during the permit procedure. Access to land was approved by all land owners.

### Study site

This study is part of the large-scale and long-term biodiversity research project ‘The Biodiversity Exploratories’ [52]. The study site was in the Schwäbische Alb Exploratory located in southwestern Germany (centroid about 48° 25′ North, 9° 26′ East) and covered 422 km^2^ [52]. Mean annual precipitation is about 700 to 1,000 mm, and average temperature is 6 to 7°C [52]. Forest patches cover 41% of the study area. The most common forest types are *Fagus sylvatica* (beech; i.e., at least 70% of the canopy is represented by beech trees with a diameter breast height ≥7 cm), mixed-deciduous (i.e., forest stands with less than 70% cover of one dominant tree species in the canopy layer), and *Picea abies* (spruce; more than 70% of spruce in canopy layer, [52]).

### Forest types and bird species

For investigating the associations among forest types, parasitism and birds' body condition, we chose 15 out of the 50 experimental forest plots. Five plots each are covered by beech, mixed-deciduous, or spruce forest, and all plots are 100 m×100 m.

We chose *F. coelebs* and *S. atricapilla* for this particular study out of the pool of 22 species available because they are the two most common species. Both species inhabit similar forest types, but they have slight differences in habitat preferences. *F. coelebs* prefers deciduous forests, which are better suited than coniferous forests [43]. Old-growth mixed-deciduous forests and forest edge habitat are considered optimal habitats for *S. atricapilla*, and highest population densities are recorded there [43].

### Capturing and handling of birds

We sampled for 51 days between April 4^th^ and July 13^th^ 2011. We sampled each experimental plot three times using 10 mist nets (8 nets of 9×2.5 m, 2 nets of 12×2.5 m). We moved mist nets during capture repetitions to minimize recaptures [53], [54]. We opened nets one hour after sunrise to hit the activity peak of birds and left the nets open for six consecutive hours. For improved capturing success of the target species, we placed three playback stations close to or under mist nets. We checked mist nets at least every 30 minutes. We followed standard field procedures for handling, measuring [53], and collecting blood. All birds were handled within 10 minutes after checking the nets.

We identified the species and sexed, aged, and banded each bird with an aluminum standard band from the *Vogelwarte Radolfzell* with a unique identification number. We determined body weight to the nearest 0.5 g and measured the length of the bill, tarsus, 3^rd^ primary counted from the outside (p3), and flattened wing from tip to bow, all to the nearest 0.1 mm, feather to the nearest of 0.5 mm. To determine the degree of morphological asymmetry for each individual, we measured all morphological traits on both sides of each individual. In addition, we noted the fat- and muscle-index [55].

We obtained blood from the brachial vein and took up to 30 µl with a micro-capillary tube [14], [56]. We prepared two thin blood smears that were air-dried and fixed in 100% methanol for five minutes. We subsequently stained them in the laboratory with Giemsa (Merck, Darmstadt, Germany) mixed in a saline buffer solution of disodium hydrogen phosphate (Na_2_HPO_4_, Suprapur®, Merck, Darmstadt, Germany) and potassium di-hydrogen phosphate (KH_2_PO_4_, EMSURE®, Merck, Darmstadt, Germany) [14].

### Parasite and leukocyte numbers

We scanned blood smears with an Axio Scope A1 microscope (Zeiss, Jena, Germany) with an integrated camera Axio Cam ICc 3 to count avian blood parasites. We first screened the entire slide at 400× to detect blood parasites [14]. We then inspected 100 visual fields on each slide with a 100× objective under oil immersion and a 10× ocular to calculate relative intensity [14]. We noted the number of the various kinds of white blood cells. We determined intensity of parasitism by counting the number of parasites per 10,000 red blood cells [14], [57], [58]. Parasites were identified following taxonomic descriptions in [14]. We identified parasites to species level whenever possible, but this proved difficult in some samples because infection intensity was low. Visual detection of blood parasites is not as sensitive as when combining microscopy and PCR [14], [30], however here we only used microscopy.

White blood cell counts allow prognosis regarding the immunoreactions and body condition of the bird [59]. A high number of lymphocytes indicate a stimulated immune system, whereas a low lymphocyte count might be correlated with immunosuppression, a viral infection, or stress [32], [59]. Therefore, to assess the immune status of birds, we distinguished and counted lymphocytes, monocytes, granulocytes (heterophils, basophils, and eosinophils), and thrombocytes [30]. The H/L-ratio indicates whether the immune system is suppressed or activated and can be used as a surrogate of chronic stress [59]. Once immune cells locate foreign bodies, such as blood parasites, the production of additional white blood cells is initiated [30]. Mainly heterophils play a crucial role in controlling bacterial, viral, and parasitic infections, because of their phagocytic capability [30]. The H/L-ratio increases with increased stress levels, but is low in vertebrates with reduced or no stress (e.g., [31]). Because white blood cell count in general is a poor indicator of stress levels, we used mainly the H/L-ratio (cf. [35]), but mention leukocyte counts for completeness throughout.

### Data analysis

We calculated prevalence (proportion of the sampled bird population that was infected [62], [63]) and intensity of parasite infection (mean number of parasites found in infected birds [63]) and we provide 95% confidence intervals for the infection parameters. We tested for significant differences among forest types for each one of the parasite population parameters; we used Mood's median test for parasite intensity and Fisher's exact test for parasite prevalence [64]. These analyses were performed with the software Quantitative Parasitology 3.0 [64].

We calculated asymmetry of the tarsi, wings, and 3^rd^ primaries (p3) as *A*_i_ = (*R*_i_−*L*_i_)/((*R*_i_+*L*_i_)/2), where *R*_i_ is the right side measurement and *L*_i_ left side measurement, as a way to gauge body development [60], [61]. We tested whether variation in parasitism is correlated with forest type, H/L-ratio, leukocyte numbers, asymmetry (p3, tarsi, wings) and hosts' sex by applying a generalized linear model (GLM with the command glm() in R [65]). We added to the basic model interaction terms for parameters that are biologically relevant, i.e., leukocytes and H/L-ratio are body condition (i.e. health status) parameters, while body asymmetries are indices for trade-offs during development. The model for parasite prevalence for example was specified as: glm(parasite prevalence ∼ H/L-ratio + (H/L-ratio * leukocytes) + leukocytes + forest-type + sex + wing-asymmetry + primary3-asymmetry + tarsus-asymmetry + (wing-asymmetry * primary3-asymmetry * tarsus-asymmetry)) and repeated for parasite intensity. We analyzed the models for *F. coelebs* and *S. atricapilla* separately.

For parasite prevalence we used a binomial distribution and for parasite intensity we used a Poisson distribution (overall models were neither over- nor under-dispersed with these settings). We also tested whether asymmetry of extremities, number of leukocytes, and H/L-ratio differed between parasitized and non-parasitized birds. We applied Wilcoxon-test or a Mann-Whitney rank sum test if the dataset was not normally distributed. All statistical analyses were performed with R version 2.13.2 [65], except as stated above.

## Results

We captured 462 birds from 22 species during 51 capture days. Among these, we drew blood from 81 *F. coelebs*, and 70 *S. atricapilla* (Table 1). *Haemoproteus* sp. caused 99.4% of infections, whereas the rest were caused by *Leucozytozoon* sp., *Plasmodium* sp., and microfilaria nematodes.

**Table 1:** Total captures, mean values and confidence levels of parasitism measures of *Fringilla coelebs* (European Chaffinch) and *Sylvia atricapilla* (Blackcap) in beech, mixed-deciduous, and spruce forest stands of the Schwäbische Alb during 2011.

Species:		*Fringilla coelebs*				*Sylvia atricapilla*		
Habitat:	Beech	Mixed-deciduous	Spruce	All	Beech	Mixed-deciduous	Spruce	All
Total captures	29	29	33	91	30	28	25	83
Infected^a^	8	6	7	21	16	16	15	47
Non-infected^a^	20	23	25	68	12	11	8	31
Total female captures	7	8	7	22	6	8	6	20
Total male captures	22	20	26	68	24	20	19	63
Mean parasite prevalence	**0.286**	*0.207*	0.219	0.231	*0.571*	0.593	**0.652**	0.566
Lower Confidence Level^b^	0.13	0.08	0.09	0.15	0.37	0.39	0.43	0.45
Upper Confidence Level^b^	0.49	0.40	0.40	0.33	0.76	0.78	0.82	0.68
Mean parasite intensity	**91.12**	28.33	*27.29*	51.90	**90.44**	72.44	*17.53*	61.04
Lower Confidence Level^c^	4.12	8.00	2.00	14.80	27.70	23.70	10.90	32.30
Upper Confidence Level^c^	432.00	60.60	101.00	200.00	217.00	210.00	23.60	115.00

Maximum values per bird species for parasite prevalence and intensity are highlighted in **bold**, whereas the smallest values are marked in *italics*.

a Infected and non-infected do not necessarily equal all captures, because several individuals were captured but not screened for parasites.

b Confidence Limits of mean parasite prevalence (Clopper-Pearson; 95% Confidence Level).

c Bootstrap Confidence Limits of mean parasite intensity (95% Confidence Level).

### Forest type and parasitism in birds

Mean parasite prevalence and parasite intensity differed between *F. coelebs* and *S. atricapilla* (Table 1); these parameters were also different within each bird species with reference to forest type (Table 1). Prevalence and intensity of parasites were highest for *F. coelebs* in beech forest stands. Parasite prevalence was highest in spruce forest for *S. atricapilla* (Table 1); nevertheless, mean parasite intensity was highest in beech and mixed-deciduous forests. Results indicated that parasite parameters (two-sided comparison) of the two species responded differently to forest type (Table 1), indicating an association of parasitism with forest type. Nevertheless, differences among forest types in parasite prevalence (Fisher's exact test, p>0.05) and parasite intensity (Mood's median test, p>0.05) were not significant within each bird species.

Regarding variation in parasite prevalence (GLM), we found a significant effect of forest type in both *F. coelebs* and *S. atricapilla* (Table 2). Wing asymmetry, tarsus asymmetry, leukocytes, and H/L-ratio had a significant effect on both species' parasite prevalence in the three forest types, while sex was additionally a significant effect for *S. atricapilla* parasite prevalence in reference to forest type (Table 2). Variation in parasite intensity of both species did not have any significant association with forest types (Table 2).

**Table 2:** Deviance analysis based on χ^2^-staistics for Generalized Linear Models (GLM) on parasite parameters of *Fringilla coelebs* (European Chaffinch) and *Sylvia atricapilla* (Blackcap) of the Schwäbische Alb in 2011.

		*Fringilla*	*coelebs*				*Sylvia*	*atricapilla*		
Parasite prevalence	DF	Deviance	Residual DF	Residuals deviance	p	DF	Deviance	Residual DF	Residuals deviance	p
H/L-ratio	1	0.001	67	0.415	**<0.001**	1	0.000	61	0.186	**<0.001**
Leukocytes	1	0.001	66	0.414	**<0.001**	1	0.000	60	0.186	**<0.001**
Forest	2	0.414	64	0.000	**<0.001**	2	0.186	58	0.000	**<0.001**
Sex	1	0.000	63	0.000	1.000	1	0.000	57	0.000	**<0.001**
Wing-asymmetry	1	0.000	62	0.000	**<0.001**	1	0.000	56	0.000	**<0.001**
Primary3-asymmetry	1	0.000	61	0.000	1.000	1	0.000	55	0.000	**<0.001**
Tarsus-asymmetry	1	0.000	60	0.000	**<0.001**	1	0.000	54	0.000	**<0.001**
H/L-ratio: Leukocytes	1	0.000	59	0.000	1.000	1	0.000	53	0.000	1.000
Wing-asymmetry: Primary3-asymmetry	1	0.000	58	0.000	1.000	1	0.000	52	0.000	**<0.001**
Wing-asymmetry: Tarsus-asymmetry	1	0.000	57	0.000	1.000	1	0.000	51	0.000	1.000
Primary3-asymmetry: Tarsus-asymmetry	1	0.000	56	0.000	**<0.001**	1	0.000	50	0.000	**<0.001**
Wing-asymmetry: Primary3-asymmetry: Tarsus-asymmetry	1	0.000	55	0.000	1.000	1	0.000	49	0.000	**<0.001**
Parasite intensity	DF	Deviance	Residual DF	Residuals deviance	p	DF	Deviance	Residual DF	Residuals deviance	p
H/L-ratio	1	0.383	67	0.254	0.536	1	0.002	61	0.943	0.968
Leukocytes	1	0.001	66	0.254	0.980	1	0.138	60	0.805	0.710
Forest	2	0.004	64	0.250	0.998	2	0.110	58	0.696	0.947
Sex	1	0.008	63	0.242	0.931	1	0.169	57	0.527	0.681
Wing-asymmetry	1	0.000	62	0.242	0.991	1	0.015	56	0.511	0.901
Primary3-asymmetry	1	0.002	61	0.240	0.961	1	0.022	55	0.489	0.883
Tarsus-asymmetry	1	0.002	60	0.238	0.968	1	0.000	54	0.489	0.998
H/L-ratio: Leukocytes	1	0.005	59	0.233	0.946	1	0.013	53	0.477	0.910
Wing-asymmetry: Primary3-asymmetry	1	0.014	58	0.219	0.905	1	0.017	52	0.460	0.896
Wing-asymmetry: Tarsus-asymmetry	1	0.024	57	0.195	0.876	1	0.023	51	0.437	0.879
Primary3-asymmetry: Tarsus-asymmetry	1	0.010	56	0.185	0.921	1	0.014	50	0.423	0.906
Wing-asymmetry: Primary3-asymmetry: Tarsus-asymmetry	1	0.098	55	0.087	0.755	1	0.005	49	0.417	0.942

Interaction terms in GLMs are indicated by double point (:) between parameter names. Significant differences (p≤0.05) are highlighted in **bold**. DF  =  degree of freedom.

### Body condition and forest type

**Figure 1:**
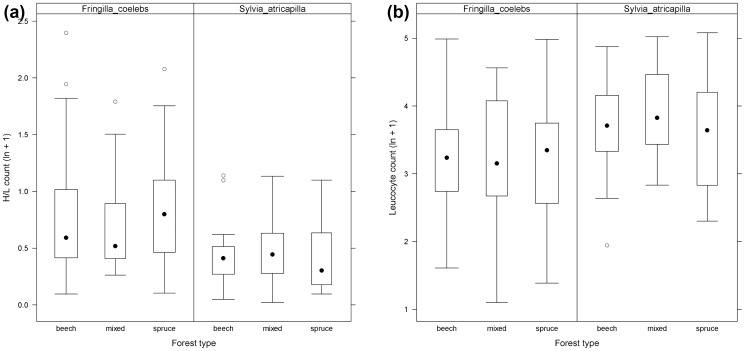
Immunoreaction parameters of *Fringilla coelebs* (European Chaffinch) and *Sylvia atricapilla* (Blackcap) in beech, mixed-deciduous, and spruce forest stands. (a) H/L-ratio, and (b) number of leukocytes per 100 visual fields. *Boxes* represent 1^st^ and 3^rd^ quartiles, *circles* outliers, *whiskers* 95% confidence intervals, and *black dots* the median.

**Figure 2:**
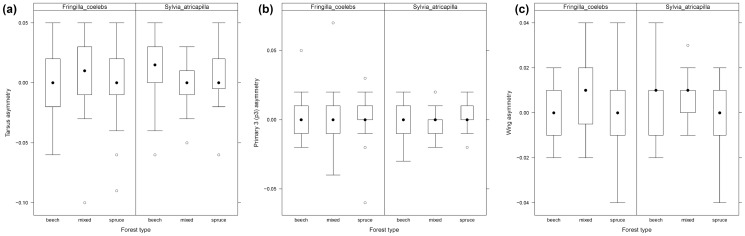
Body asymmetries of *Fringilla coelebs* (European chaffinch) and *Sylvia atricapilla* (blackcap) in beech, mixed-deciduous, and spruce forests stands. (a) Tarsus asymmetry, (b) primary 3 asymmetry, and (c) wing asymmetry. A value of 0 indicates symmetry of the individuals, and any value thereof indicates asymmetry to one side. *Boxes* represent 1^st^ and 3^rd^ quartiles, *circles* outliers, *whiskers* 95% confidence intervals, and *black dots* the median.

We found differences between the H/L-ratio (Figure 1a), leukocytes (Figure 1b), and asymmetries (Figure 2) in reference to the three forest types. We found a non-significant increase in leukocyte numbers in mixed-deciduous forests and an increased H/L-ratio (Figure 1a) in spruce forests for *F. coelebs*. Lymphocytes did not differ between the three forest types (Figure 1b). The H/L-ratio was highest for *S. atricapilla* sampled in mixed-deciduous forests (χ^2^ = 1.67 df = 2, p>0.05). Overall, *S. atricapilla* had lower H/L counts than *F. coelebs*.

Asymmetries differed regarding forest types for both species (Figure 2). *F. coelebs* was more or less symmetric in beech and spruce forests, but skewed in mixed-deciduous forests; *S. atricapilla* had larger asymmetries in beech forests (Figure 2). Tarsus and wing asymmetries (primary 3 only for *S. atricapilla*; cf. Table 2) had also significant associations (GLM) with variance in parasite prevalence.

### Parasitism and body condition

We found no significant differences in asymmetries between infected and uninfected individuals in both species, regardless of forest types. The H/L-ratio was significantly higher in infected compared to uninfected *S. atricapilla* (Wilcoxon-test; W = 387.5, p<0.05), but not for *F. coelebs* (Mann-Whitney: T = 930, p>0.05). Total number of white blood cells (Figure 1b) was significantly higher in infected than uninfected birds (*S. atricapilla*: Wilcoxon-test, W = 350; p<0.05; *F. coelebs*: Mann-Whitney: T = 1,017.5, p = 0.03), suggesting an immunoreaction of birds to infection.

## Discussion

### Forest type and parasitism

Contrary to our expectations, we found that all parasite population parameters were higher for both bird species captured at beech and mixed-deciduous forests, except for prevalence in *S. atricapilla*, which was higher in spruce forests. Because both species respond differently in body condition to parasitism and forest types, we suggest two possible mechanisms: (i) forests with favorable habitat characteristics are preferred by host individuals thereby increasing intra-specific interactions in these habitats [5]. Hence, individuals occupying a territory in high quality habitats might have to compete with more rivals than a bird occupying suboptimal habitats [4], leading to higher time and energy investment in defending their territory. These extra costs possibly reduce the individual's body condition, increase stress, and augment susceptibility to infection [66]–[72]. (ii) This process can also happen in reverse, by birds already occupying suboptimal habitats facing scarcer resources, leading to higher time and energy investment which in turn leads to extra costs and higher susceptibility to infection. In both species, we find a common association with habitat, suggesting that vector transmission is differential and it would be higher in beech or mixed forests.

One single highly parasitized individual caused the increased parasite intensity measured in *F. coelebs* from beech forest. Such differences in the number of parasites per individual are attributable to the natural way in which parasites are distributed in the environment (following a negative binomial distribution, see [72]–[74]), in which a few individuals have a large number of parasites, and most hosts have only a few parasites [73]. Overall, the associations between parasites and forest types have detectable signals in our data, but potential causal relationships between parasites and forest types remain open.

### Forest type and body condition

Neither bird species showed significant increased stress levels on the Schwäbische Alb in reference to forest type. We assessed the H/L-ratio because this index offers evidence of long-term stress in birds [31]–[34]. Even if changes in the H/L-ratio can occur rapidly while handling birds [35], [75], the H/L-ratio can be used as a good indication for immunoreaction to elevated stress levels [35], [36]. Since we could not standardize time of drawing blood after capture and leukocyte counts can rapidly change with handling [75], differences on handling time may affect the results of leukocyte counts. The H/L-ratio did vary, however, and was highest in spruce (*F. coelebs*) or mixed-deciduous (*S. atricapilla*) forest, indicating higher stress levels for each bird species in different habitat types. While we expected higher stress levels for both species in spruce forests, *S. atricapilla* does exhibit higher stress levels in mixed-deciduous forests.

Although beech and mixed-deciduous forests should offer a more suitable habitat for the two species per se [18], [43], our findings indicated that *S. atricapilla* show higher stress-levels in mixed-deciduous forests. The landscape on the Schwäbische Alb is characterized by small-scale patchiness (forest stand size of 0.1 ha to 3 ha, with most < 1 ha in size) of various forest types [52]. Because the two studied passerines have territories typically between 0.1 and 2 hectares [43], the captured birds might have foraged in the different forest types across the landscape. If individuals forage in different habitats, we would not expect differences in body conditions since the habitat effects would level off. This suggests, that the differences we found are not a foraging effect but driven likely by the habitat type.

In addition, migrant species such as *S. atricapilla* use habitat on migration which might be the source of infection; the infections obtained in stop-over or wintering habitats can be carried-over to the breeding habitat and the infected birds might be forced to use suboptimal habitats on return because of their reduced body condition and limited ability to select suitable habitat patches. Use of various forest types for foraging might be a way to compensate for low food supply within one forest type (e.g., spruce forest is less suitable per se for both species; [43]), providing that movement does not represent long distances with high-energy expenditure and elevated predation risk.

The wing, tarsus, and primary 3 asymmetries of *S. atricapilla* and *F. coelebs* differed among forest types. This might represent evidence that beech and spruce forests hold healthier individuals than mixed-deciduous forests for *F. coelebs*, particularly considering that sample size is about equally high in all three habitat types. For *S. atricapilla*, we found significant differences regarding beech, indicating equally suitable habitat conditions for the species in the other two types. Some studies investigating fluctuating asymmetries have successfully explained the effects of environmental stress factors [76]–[78]. Recently, a study [50] showed that parasite infections could indeed have an effect on body asymmetries. Asymmetries of wing and primary 3 develop during the molt (about July/August), while tarsus growth occurs during juvenile development on the nest. Therefore, both imply different times when parasitism or stress occurred, namely during development of the morphological parameter [50]. The association of asymmetries with habitat and parasitism might be relatively loose, but nevertheless, associations during asymmetric development might be given for the relevant time frames when the asymmetries are developed [60], [61].

### Parasitism and body condition

In addition to the influence of forest type on the response of the health status of birds to parasitism, we were interested in general effects of a parasitic infection on body condition parameters. Hence, we compared parasitized versus non-parasitized birds regarding fluctuating asymmetries, H/L-ratio, and total white blood cell count.

If a bird suffers from a blood parasite infection, the number of heterophils increases to fight infectious agents (heterophilia; [30]). This might explain the high H/L-ratio that we have found in the blood of infected birds. Yet, corticosteroid release during stressful conditions might also elevate heterophil numbers. However, stressors such as short transport of less than one hour are not likely to influence white blood cell counts [35], [36], [79]. Thus, we conclude that any increase in leukocyte and, especially, heterophil numbers is attributable to parasite infection and not due to handling.

The total number of white blood cells was significantly increased in parasitized birds, indicating that *F. coelebs* and *S. atricapilla* respond to an infection by activating immune cells. We found no significant differences of the H/L-ratio between parasitized and non-parasitized *F. coelebs*, but significant differences were apparent for *S. atricapilla*, suggesting that these two species have different stress responses to infection (H/L-ratio is a well-suited indicator of stress [31]–[34]). Furthermore, parasitized and non-parasitized birds had the same body condition, which might be attributable to the present phase of infection or simply to the heterogeneity of the effect that different parasite species have on different bird species and individuals (cf. [80]–[82]). In addition, parasite species infecting *F. coelebs* and *S. atricapilla* might have different pathologic effects on either host species (cf. [14], [30]). During an infection with haemosporidian parasites, a prepatent period occurs (parasites develop within tissues and are not yet detectable in peripheral blood), followed by an acute phase in which the number of parasites in the peripheral blood reaches a peak [14]. If the immune system of a bird is strong enough to fight the infection, such an infection becomes chronic and perhaps can be cleared, but normally, a blood parasite infection persists for the whole life of a bird [14]. In this study, the examined birds mainly suffered from light infections, which we interpret as their immune system being able to reduce the number of blood parasites to a low level. Thus, these individuals might recover from the acute phase of infection, and any effects on fitness measurements (i.e., weight and body condition index) are no longer detectable (see [80]).

Taken together, our results suggest that haemosporidians do not have strong effects on the general body condition of the two studied bird species, but that they stimulate an immune response [30], [80]. Our results also indicate that host-parasite interactions are affected by the hosts' habitat type; taking the role of dipteran vectors into account might improve the causal link between parasites and forest type.
